# Willingness to pay for an mHealth anti-retroviral therapy adherence and information tool: Transitioning to sustainability, Call for life randomised study experience in Uganda

**DOI:** 10.1186/s12911-022-01782-0

**Published:** 2022-02-26

**Authors:** Agnes Bwanika Naggirinya, Eunice L. Kyomugisha, Maria S. Nabaggala, Benson Nasasira, Josephine Akirana, Elizabeth Oseku, Agnes Kiragga, Barbara Castelnuovo, Rachel L. King, Elly Katabira, Dathan M. Byonanebye, Mohammed Lamorde, Rosalind Parkes-Ratanshi

**Affiliations:** 1 Infectious Diseases Institute, College of Health Sciences, Makerere University, Hall Lane, P.O. Box 22418, Kampala, Uganda; 2 University of California, San Francisco, USA; 3 Department of Internal Medicine, College of Health Sciences, School of Medicine, Makerere University, P.O. Box 7062, Kampala, Uganda; 4 University of Cambridge, Cambridge, UK

**Keywords:** Sustainability, mHealth, HIV, ART, Call for life, Payment evaluation

## Abstract

**Introduction:**

Evidence shows benefit of digital technology for people living with human immunodeficiency virus on antiretroviral therapy adherence and retention in care, however, scalability and sustainability have scarcely been evaluated. We assessed participants’ willingness to pay a fee for mHealth “Call for life Uganda” support, a mobile-phone based tool with the objective to assess sustainability and scalability.

**Methods:**

“Call for Life study”, approved by Makerere University, School of Public Health research & ethics committee, at 2 sites in Uganda, evaluated a MoTech based software “CONNECT FOR LIFE™” mHealth tool termed “Call for life Uganda”. It provides short messages service or Interactive Voice Response functionalities, with a web-based interface, allows a computer to interact with humans through use of voice and tones input via keypad. Participants were randomized at 1:1 ratio to Standard of Care or standard of care plus Call for life Uganda. This sends pill reminders, visit reminders, voice messages and self-reported symptom support. At study visits 18 and 24 months, through mixed method approach we assessed mHealth sustainability and scalability. Participants were interviewed on desire to have or continue adherence support and willingness to pay a nominal fee for tool. We computed proportions willing to pay (± 95% confidence interval), stratified by study arm and predictors of willingness to continue and to pay using multivariate logistic regression model backed up by themes from qualitative interviews.

**Results:**

95% of participants were willing to continue using C4LU with 77.8% willing to pay for the service. Persons receiving care at the peri-urban clinic (OR 3.12, 95% CI 1.43–9.11.86) and those with exposure to the C4LU intervention (OR 4.2, 95% CI 1.55–11.84) were more likely to continue and pay for the service. Qualitative interviews revealed mixed feelings regarding amounts to pay, those willing to pay, argued that since they have been paying for personal phone calls/messages, they should not fail to pay for Call for life.

**Conclusions:**

Payment for the service offers opportunities to scale up and sustain mHealth interventions which may not be priorities for government funding. A co-pay model could be acceptable to PLHIV to access mHealth services in low resource settings.

*Clinical Trial Number* NCT 02953080.

**Supplementary Information:**

The online version contains supplementary material available at 10.1186/s12911-022-01782-0.

## Introduction

In 2018, 37.9 million people were living with human immunodeficiency virus (HIV) globally, with 23.3 million accessing antiretroviral therapy (ART) [1]. The World Health Organization (WHO) 2016 guidelines emphasize the need for strong systems to link patients in care and to strengthen ART adherence, and long-term retention in care for people who initiate ART [2].

The Uganda Ministry of Health Consolidated guidelines for prevention & treatment of HIV, emphasizes pre-ART adherence counselling before initiating ART and ongoing adherence support thereafter. Counselling can be “Provider- initiated” or “Client-initiated”. The same guidelines list mobile phone calls & text messages as part of adherence support interventions [3].

The introduction of information and communication technologies (ICT) in healthcare, especially the application of mobile communications, is transforming healthcare delivery making it more accessible, affordable and available [4]. Mobile technologies could be a powerful media for providing individual level support to health care consumers [5] and strengthen health systems in resource limited settings [6]**.**

Patient-centered mobile-health approaches can improve ART adherence and promote viral load (VL) suppression in people living with human immuno deficiency virus (PLHIV) [7]. mHealth tools can support HIV patient management through: medication-adherence, clinic-attendance, health tips and retention in care in the underserved, vulnerable populations to meet the UNAIDS 90:90:90 target [8, 9].

The high mobile phone penetration rates in Sub-Saharan Africa [10] offer opportunity of using mobile health (mHealth) interventions of improving adherence to antiretroviral therapy (ART) and retention in care. Although there is suggestive evidence of benefit of digital technology for HIV care on ART adherence and retention in care [8, 11] there are concerns about scalability and sustainability of these interventions [12]. Within a randomized controlled trial, we assessed participants’ willingness to pay a fee and recommendation for mHealth “Call for life Uganda” (C4LU) support, a mobile-phone based pill reminder, appointment reminder and health message tool for chronic illnesses with the objective to assess sustainability and scalability.

## Methods

This work was embedded in a larger study entitled ‘improving outcomes in HIV patients using mobile phone based interactive software support’ “Call for life Uganda(C4LU)” https://clinicaltrials.gov/ct2/show/NCT02953080. C4LU was a randomized clinical trial at 2 sites, the Infectious Diseases Institute (IDI) which is an urban center of excellence in HIV care, and Kasangati Health Center, which is a peri-urban public health care facility https://mhealth.jmir.org/2021/2/e22229/. The primary outcome were to determine the impact of interactive voice response (IVR) technology on Medical Outcomes Study HIV quality of life ( QoL) scores and viral suppression at 12 months [13]. The intervention “technology” evaluated in this study was based on CONNECT FOR LIFE™ (CfL) m-health technology. Janssen, the Pharmaceutical Companies of Johnson & Johnson developed CONNECT FOR LIFE™ m-health technology as a community health information technology platform for health initiatives to assist patients in developing countries. The intervention arm, high users (picked > 75% of pill reminders) had overall higher QoL compared to low users (picked < 25% of pill reminders) (92.2 versus 87.8, *P* = 0.02). High users had higher QoL scores in the mental health domain (93.1 versus 86.8, *P* = 0.008) and better appointment keeping. Similarly, participants with moderate use (51%-75%) had better viral suppression at 12 months [13].

CfL is a mobile communication platform which interacts with patients using basic mobile phone technology and with healthcare providers through a web-based interface, with text message or Interactive Voice Response (IVR) functionalities. IVR allows a computer to interact with humans through the use of voice and tones input via keypad.

CfL offers individualized pill reminders, visit reminders, health tips and functionality to support symptom reporting (see Additional file 1: Appendix). It was adapted for use in PLHIV in Uganda by IDI and termed “Call for Life” (C4LU).

### Data collection

Through mixed methods data collection at study exit, quantitative data was obtained through an interviewer administered exit questionnaire (Additional file 2: Appendix) about desire to have adherence support and willingness to pay a nominal fee for C4LU. The objective for the interview was to assess mHealth sustainability and scalability. The exit questionnaire had 11 questions, with four strategies to assess: “User-pay-for–service”, “User-payment-mode”, “User-payment-schedule”, “User recommendation to a colleague or other chronic disease” and “frequency of adherence support”. Frequencies and odds ratio with 95% confidence levels were calculated. We determined the predictors of willingness to continue and to pay using multivariate logistic regression model.

For the qualitative part, a purposive sampling of 55 participants from the intervention arm was recruited through a telephone call by one lead social scientist and a research assistant based on categories of main study participants which were not mutually exclusive (positive partners in HIV sero-discordant relationships, young adults 18-25yrs, PLHIV established on 1st or 2nd Line ART, PLHIV Initiating on 1st Line ART, breastfeeding mothers, and most at risk populations (MARPS). All participants gave written informed consent.

Lead social scientist completed face- to-face semi-structured interviews with focus groups of 8–12 participants. Recruitment of participants continued until no new themes emerged from analysis. Data was analyzed using a thematic analysis and content analysis framework while coding key and emerging issues for further analysis. The distribution of themes across key populations was also examined and was supported by quotes based on their thematic similarities. Key themes explored included: Willingness to pay for CFLU services, why clients should pay for the services, why clients should not pay for the services, mode of payment, amount to pay and suggestions to raise income to meet payment costs.

### Statistical methods

Described participant’s characteristics using medians (interquartile range, IQR) and frequency distributions. Logistic regressions model was used to establish factors associated with willingness to receive or continue with (those on intervention) mHealth adherence support and willingness to pay for sustainability of mHealth adherence support adjusting for gender, age, ARM, site, having a spouse, education level, duration on ART, and employment status. All *P*-values were considered significant if *P*-value < 0.05. Analysis was performed using Stata version 13.0 (StataCorp, College Station, Texas, USA).

### Ethical review

Trial approval was received from Makerere University School of Public Health, Higher Degrees Research & Ethics Committee (Number: 378) and research clearance from Uganda National Council of Science & Technology (UNCST Folio Number**:** *HS 3005*) ClinicalTrials.gov (Reg number: NCT 02953080). The protocol was performed in accordance to UNCST guidelines and regulations.

## Results

**Fig. 1:**
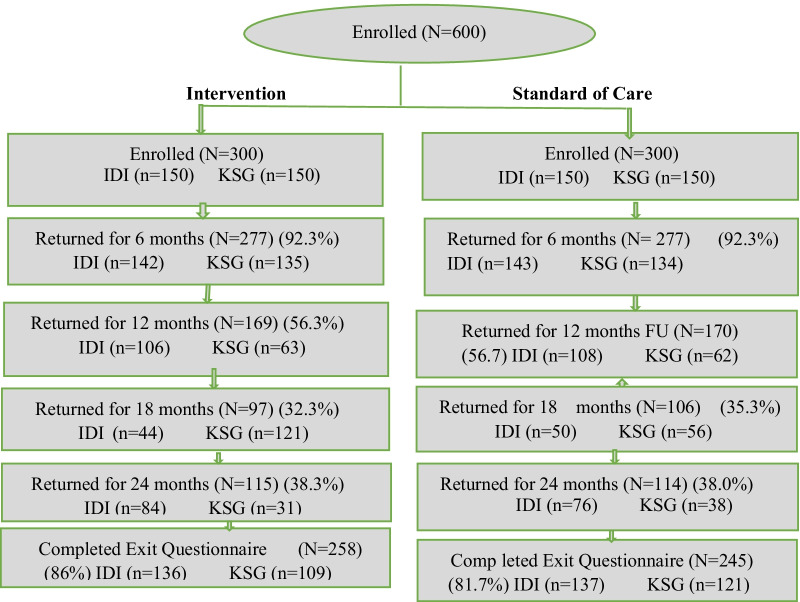
Study flow diagram. Shows number of participants at various visit follow-up for the main study and final number that completed the exit willingness questionnaire

Of 600, 503 completed the exit questionnaire; 89 did not complete study (49 early withdraw/termination from study, 40 did not turn-up for the study exit visit), 8 did not complete the filling in of exit questionnaire. Of the 503 who completed the questionnaire, 245 (48.7%) were on C4LU intervention arm and 258 (51.3%) on Standard of Care arm. Figure 1 shows the numbers that completed the exit questionnaires at 12, 18 and 24 (see Fig. 1).

Table 1, shows the demographics of the 503 participants that completed the exit questionnaire; over half of the participants belong to the Infectious Diseases Site, majority were females (67%), over 50% were 35yrs and below. A total of 384 (76.3%) participants had spouses/partners and over half of the study population attained secondary plus education level, a third of the study population was unemployed and median duration on ART was 2.9 years.

**Table 1:** Demographics by ARM for participants who completed the exit questionnaire

Variables	Standard of Care arm N = 245(%)	Interventional arm N = 258 (%)	Total N = 503
Study site			
Infectious Diseases Institute-Mulago	137(53.1%)	136(55.5%)	273(54.3%)
Kasangati Health Centre IV	121(46.9%)	109(44.5%)	230(45.7%)
Gender			
Female	173(67.1%)	164(66.9%)	337(67.0%)
Male	85(32.9%)	81(33.1%)	166(33.0%)
Age (years)			
16–24	62(25.3%)	62(24.0%)	124(24.7%)
25–35	101(39.2%)	83(33.8)	184(36.6%)
36–50	77(29.8%)	83(33.8%)	160(31.8%)
51+	18(6.98%)	17(6.9%)	35(7.0%)
Currently having a partner/spouse			
Yes	199(77.1%)	185(75.5%)	384(76.3%)
No	59(22.9%)	60(24.5%)	119(23.7%)
Highest education level			
None	12(4.7%)	7(2.9%)	19(3.8%)
Primary	96(37.21%)	95(38.8%)	191(38.0%)
Secondary	109(42.3%)	111(45.3%)	220(43.7%)
Tertiary	41(15.9%)	32(13.1%)	73(14.5%)
Employment status			
Yes	181(70.2%)	170(69.4%)	351(69.8%)
No	77(29.8%)	75(30.6%)	152(30.2%)
ART duration in years median (IQR)	2.7 (0.5–5.2)	3 (0.5–5.2)	2.9 (0.5–5.4)

Table 2, shows proportions willing to continue with receiving mHealth support and those willing to pay for it, payment modality and schedule. Majority (95%) of the participants were willing to continue with the support, and over 70% were willing to pay a fee.

**Table 2:** Participants’ responses regarding willingness to pay, payment modes, and frequency for 503 participants

Variables	Intervention	Standard	Total	Chi-square	*P*-value
Willing to continue support					
No	5 (2.0)	20 (7.7)	25 (5.0)	8.678	0.003
Yes	240 (98.0)	238 (92.3)	478 (95.0)		
Willing to pay for the support					
No	47 (19.6)	59 (24.8)	106 (22.2)	1.877	0.171
Yes	193 (80.4)	179 (75.2)	372 (77.8)		
Payment mode					
Mobile money	87 (46.0)	94 (53.7)	181 (49.7)	2.145	0.143
Airtime	102 (54.0)	81 (46.3)	183 (50.3)		
Frequency of payment					
Daily	25 (12.9)	25 (15.1)	50 (14.0)	8.548	0.073
Weekly	42 (21.8)	44 (25.1)	86 (23.4)		
Monthly	117 (60.7)	91 (49.2)	208 (55.1)		
Yearly	9 (4.7)	19 (10.6)	27 (7.3)		
Recommendation to a Colleague					
No	1 (0.4)	5 (1.9)	6 (1.2)	2.495	0.114
Yes	244 (99.6)	253 (98.1)	497 (98.8)		
Government adopt it for chronic illnesses (hypertension, diabetes, tuberculosis)					
Hypertensive					
No	133 (54.3)	145 (56.2)	278 (55.3)	0.186	0.666
Yes	112 (45.7)	113 (43.8)	225 (44.7)		
Diabetes					
No	120 (49.0)	135 (52.3)	255 (50.7)	0.563	0.453
Yes	125 (51.0)	123 (47.7)	248 (49.3)		
Tuberculosis					
No	88 (35.9)	124 (48.1)	212 (42.1)	7.600	0.006
Yes	157 (64.1)	134 (51.9)	291 (57.8)		

Over 50% mentioned mobile money as the payment mode and majority (55%) requested to pay monthly.

Table 3, shows the regression models for continued mHealth support, the participants who were exposed to mHealth were 4 times likely to continue with the support, and the participants in the peri-urban Health Centre were 4 times like to continue with support compared to those at Infectious Diseases Institute Clinic (IDC), which is an Urban Centre of excellence in HIV care.

**Table 3:** Factors associated with willingness to receive or continue with (those on intervention) mHealth adherence support

	Un-adjusted model			Adjusted model		
Variable	OR	95% CI	*P*-value	OR	95% CI	*P*-value
Gender						
Female	1			1		
Male	1.59	0.62–4.06	0.330	2.03	0.67–6.16	0.210
Age						
16–24	1					
25–35	1.34	0.50–3.58	0.558	1.35	0.46–3.93	0.58
36–50	1.77	0.59–5.24	0.303	1.53	0.43–5.50	0.51
51+	1.13	0.23–5.62	0.874	0.79	0.12–5.30	0.82
Study arm						
Standard of care	1			1		
Intervention	4.03	1.49–10.9	0.006	4.28	1.5–11.85	0.005
Study site						
IDC Mulago	1			1		
Kasangati H C IV	2.79	1.09–7.12	0.031	4.13	1.44–11.86	0.008
Currently have spouse/partner						
Yes	1			1		
No	0.64	0.27–1.53	0.32	0.85	0.33–2.18	0.74
Highest level of education						
None	1			1		
Primary	1.12	0.13–9.40	0.91	1.043	0.12–9.8	0.97
Secondary	1.17	0.14–9.63	0.88	1.26	0.14–11.13	0.83
Tertiary	0.76	0.08–6.88	0.80	1.14	0.11–11.4	0.911
Currently employed						
Yes	1			1		
No	0.92	0.38–2.17	0.84	0.92	0.35–2.4	0.860
Antiretroviral therapy duration	1.016	0.89–1.16	0.800	1.017	0.94–1.094	0.658

Table 4, shows regression models for mHealth payment, the participant on mHealth intervention were almost 2 times willing to pay compared to those on standard of care.

**Table 4:** Factors associated with willingness to pay for sustainability of mHealth adherence support

	Un-adjusted model			Adjusted model		
Variable	OR	95% CI	*P*-value	OR	95% CI	*P*-value
Gender						
Female	1			1		
Male	1.17	0.76–1.80	0.48	1.40	0.83–2.38	0.206
Age						
16–24	1			1		
25–35	1.62	0.95–2.76	0.078	1.72	0.98–3.02	0.059
36–50	0.95	0.56–1.59	0.83	0.87	0.47–1.60	0.654
51+	0.98	0.43–2.56	0.968	0.79	0.31–2.08	0.654
Study arm						
SoC	1			1		
Intervention	1.56	1.03–2.33	0.035	1.63	1.1–2.5	0.022
Study site						
IDC Mulago	1			1		
Kasangati HCIV	1.16	0.77–1.74	0.470	1.32	0.81–2.14	0.259
Currently married						
Yes	1			1		
No	1.22	0.75–1.98	0.430	1.34	0.80–2.23	0.259
Highest level of education						
None	1			1		
Primary	1.01	0.35–2.94	0.99	1.035	0.34–3.12	0.951
Secondary	1.07	0.37–3.11	0.89	1.099	0.365–3.31	0.866
Tertiary	1.09	0.35–3.45	0.88	1.057	0.33–3.64	0.869
Currently employed						
Yes	1			1		
No	0.85	0.55–1.31	0.459	0.84	0.515–1.36	0.478
ART duration	0.988	0.928–1.052	0.717	1.02	0.94–1.094	0.658

The amount of fees which the individual participants were willing to pay, and the frequency of payment: Majority of individuals wished to pay a monthly fee of 1.35USD which is equivalent to 5000 Uganda Shillings (UGX), while a few mentioned an annual payment of 13.5 USD which is equivalent to 50000UGX.

Out of 600 participants, a total of 503 completed the questionnaire, 245 on C4LU intervention arm while 258 on SoC arm, 89 did not complete study due to various reasons (49 had been terminated/withdrawn early, 40 did not turn-up for the close-out visit), 8 did not complete the exit questionnaire. The questionnaire asked on type of adherence support the participant was getting; face-to-face facility based 6 monthly adherence support (standard of care) or mHealth daily adherence support in addition to SoC, continuity of support, frequency, recommendation of services to colleagues or any chronic care illness stating reasons for any answers given. Before assessment for willingness to pay, the questionnaire asked the participants to rate the services at a Likert scale 1–5, with 1 being poor and 5 excellent.

Overall, 478/503 (95.03%) wanted continued adherence support and 375 (78.45%) were willing to pay for C4LU. On the C4LU arm, 240 (97.96%) wanted to continue C4LU and 168/240 (70.00%) were willing to pay. On SOC arm, 238 (92.22%) wanted continued adherence support, and 157(65.97%) were willing to pay for C4LU.

Receiving care from a peri-urban clinic (OR 3.12, 95% CI 1.43–9.11.86) and on the C4LU intervention arm (OR 4.2, 95% CI 1.55–11.84) predicted willingness to continue and pay. Willingness was not affected by education level, employment, ART duration, gender, age or marital status [14].

### Willingness to pay for the system: qualitative findings

Qualitative findings about C4LU benefits mentioned at baseline and close-out interviews are clear pointers to the willingness in paying for the C4LU system. Despite the mixed feelings regarding specific amount to pay from participants (Discordant couples, Young adults, established 1st line and 2nd line, breastfeeding mothers, 1st line Initiating and MARPS, these groups were not mutually exclusive) (Table 5), those willing to pay, supported their responses by arguing that they have been paying for their personal phone calls and messages, and should not fail to pay for the C4LU system.

“… the way we have been paying for airtime for our phones calls.., we should be able to pay for the C4LU system” **FGD with Discordant couples – Close-out).**

**Table 5:** Thematic presentation of participants perspectives to sustainability of mHealth adherence support

Client categories (these groups were not mutually exclusive)						
	Clients established on 1st or 2nd line ART (8 participants)	Mothers on PMTCT (12 Participants)	Young adults 18–24 years (10 participants)	Clients initiating 1st or 2nd line ART—(5 participants)	Most at Risk Populations (MARPS) (9 participants)	Positive partners in discordant relationship (11)
*Main theme*						
Willingness to pay	Most willing to pay (80%)	Most NOT willing to pay (80%)	Most willing to pay (80%)	Most NOT willing to pay (80%)	All willing to pay (100%)	All willing to pay (100%)
*Sub/emerging themes*						
Payment modes	Mobile phone airtime deductions	Not reported	Mobile phone airtime deductions	Mobile phone airtime deductions	Using mobile money Mobile phone airtime deductions	Mobile phone airtime deductions
Frequency of payment	Monthly deductions	Monthly deductions	Monthly, annually	Every 3 months	Weekly, monthly	Every day, monthly
Amount to be paid (USD)	13.5ȼ, 16.2ȼ and 2.7$ monthly and	1.35$ monthly	1.35$ monthly and 8.1 $–27.0$ annually	− 27.0ȼ to 13.5 $	13.5ȼ weekly, monthly	1.35ȼ–18.9 ȼ daily and 4.1 ȼ–27.0ȼ monthly
Why they should pay	C4LU been of benefit to them	Not reported, majority did not support the idea	Pay for useless things like lip-stick, so should pay for C4LU Been of benefit to health	Not reported, majority did not support the motion	Before C4LU, were initially badly off so deserve to pay If nutritional tips are added, they’d pay	Some already pay for health insurance and so can pay for C4LU
Why they should not pay	Little earnings	Cannot afford Some even lack transport to appointments	None opposed	Cannot afford Some even lack transport to appointments Government already deducts a lot of tax from them	None objected	None objected

During the exit interview, a participant suggested to staff to determine and fix the amount to be paid. There were different views on the modes of payment both during the Close-out group discussions and dissemination that ranged from 0.0135 USD, to 0.270 USD per day. Other participants opted for weekly deductions ranging from 0.0135USD, to 1.89USD, while others suggested monthly deductions ranging from 0.135USD to 2.70 USD. Each group had a suggestion about paying for the system and women in particular were vigilant.

Some expressed the concern that stopping C4LU would distress them as they have been attached to its daily reminders, and suggested forming a savings scheme so that the proceeds from the contribution meet the cost for the entire group.

“…… may one day end due to certain circumstances like finances, we don’t know, so if it’s abruptly cut off when we have been using it, we are not sure… stopping may distress us……. when you just stop and find that C4LU is no more…… **(FGD with MARPS, – IDI Kasangati – Close-out)**

The findings on willingness to pay for the C4LU system are indicative of the value and trust that patients have attached to the system. Findings project the possibilities of C4LU sustainability.

Qualitative findings about participants’ benefits from both the baseline and close-out are clear pointers to the willingness that most participants showed in paying for the C4LU system. Emerging themes rotated around why they should pay, why they should not pay, modes of payment, amounts to pay and suggestions to raise income to meet payment costs.

### Why clients should pay for C4LU system

Some participants felt that since they have been paying for their personal phone calls and messages, they should not fail to pay for the C4LU system. Other clients’ willingness to pay was about affordability. Young adults expressed concern that some husbands do not allow their wives to work; women have to wait to save from home upkeep money and even fail to buy the required home stuff; the amount to pay should therefore be affordable to all. At close-out, a MARPS client expressed fear that when the system stops due to lack of funds, it will have a very bad impact on them, they will get embarrassed and their happiness will suddenly end, he concluded.

“Just the way we have been paying for airtime for our phones calls, the same way; we should be able to pay for the C4LU system”. **FGD with Discordant couples – Close-out, FGD with 1st and 2nd Line on ART Established – IDI Mulago – Close-out).** In a chorus, most participants similarly agreed.

“……this may be a temporary thing and may one-day end due to certain circumstances like finances, we don’t know, so if it abruptly ends when we have been using it, we are not sure… stopping may embarrass because you are happy and you do not know what it involves when you just stop and find that C4LU is no more**…. (FGD with MARPS, – IDI Kasangati – Close-out).**

Willingness to pay for the C4LU system was further re-echoed when participants compared the proposed payments with government OTT deductions of 0.5% for withdrawing money as well as spending money on useless things like pornography, mirrors, sprays and others. A participant gave a condition that if nutritional tips were included into the system, he would pay for C4LU while a discordant participant was of the view that if the amount is fair enough, affordable and will not affect his budget, he would even pay for others.

“What about this thing that helps you from day one till you die, if you continue with it……even if it is 6.4USD”. “We are paying for something that will help us move forward. If I can pay for useless things like pornography, eye pencil, lipstick, mirror, sprays and others, why wouldn’t I pay for my life……. i suggest we pay for these calls? (**Young adults—IDI Kasangati – Close-out).**

### Why clients should Not pay for C4LU system

Some clients did not buy the idea of paying for the system, these were 156 out of the 503 participants. Their argument was that they often fail to raise transport money as little as half a dollar (USD) for the clinic visits. Some people miss their appointments due to lack of transport money and which means they may not have the capacity to pay for the system, a participant explained. Other participants felt that since government deducts so much tax from them, C4LU should continue offering them free services.

“For all these things C4LU has done for us; I request that we should not pay any money for the services because there is a time I missed picking my medicine due to lack of transport. Things are not easy but we want to continue being healthy so that life moves on. If we were to start paying money, then we would be affected”. **FGD with Breastfeeding mothers (PMTCT) – IDI – Kasangati – Close-out).**

“The government deducts a lot of tax from us, let C4LU continue being free because we shall end up paying for everything. If we are to pay, may be it would be 1000” **(FGD with 1st Line and 2nd Line – Initiating – Kasangati – Close-out).**

A participant rejected all suggestions for paying for C4LU system as he queried whether the research was for profit or sponsored and wondered whether payments would not discourage others from joining. The willingness to pay was also dependent on how much to pay and how much people were earning, participants noted. They proposed that C4LU should not make life hard for them; they should continue paying for them since some of them take a while to load airtime because of government taxes.

#### Mode of payment

Several suggestions on modes of payments during close-out discussions and dissemination were made, where most participants felt they could pay indirectly by deduction from airtime, automated with draw from mobile money accounts *and* the deduction could be done weekly; monthly or after every three months:

“They had ever asked us about it and so I gave my opinion that they can deduct like 0.21USD a week from our mobile money and keep us on the system. Personally, cutting me off the system would leave a wound on me” **(FGD with Discordant Couples - IDI Mulago – Close out).**

#### Amount to pay

Participants suggested amounts that varied and ranged from 0.011 USD to 0.21USD daily, those who opted for weekly deductions suggested 0.135USD to 1.5 USD, while monthly deductions ranged from 0.11USD to 2,13USD. Some few participants requested annual pay and they suggested a fee of 13.5USD. Women in particular were vigilant in suggesting the amounts to pay.

“This thing should be done monthly. If I pay 0.021 USD, I will know that I am covered for a month. Instead of deducting like 0.15 USD every week when you have loaded airtime of 500 shs with an intention of subscribing to 6 min’ airtime, they rather deduct our money monthly” **(FGD with Discordant couples – IDI Mulago – Close-out).**

“About 1.28USD a month. It depends on how helpful and relevant their services are to me and as long as it is not more than 2.13USD. We should first look at the things we have benefited from CFLU”. **(FGD with 1st Line and 2nd Line on ART – Established – Close- Out).**

### Suggestions to raise income to meet payment costs

At close-out dissemination, some clients were of the view that it would be useful to form a local savings scheme so that proceeds from their contributions meet the cost of calls for the entire group. However, there were concerns about who would meet the costs of service fee incase the patients lost pin codes. This was clarified that the study would meet such costs in case the suggestion of cost sharing was to be implemented. The findings on willingness to pay for the C4LU system are indicative of the value and trust that patients have attached to the system. A few participants expressed inability to pay for the system; nevertheless, findings project the possibilities of C4LU sustainability; should majority patients finally afford the payments that will be later be determined.

## Discussion on sustainability

SUSTAINABILITY: There are several barriers for the outreach interventions implementation and scale-out, among which, lack of programme sustainability [15] is a major stumbling block. This barrier posed a potential harm for the intervention, as the termination of the intervention would set back ART adherence. The second barrier is the intervention not being well integrated into the existing welfare and social support and existing health system [16].

Despite hundreds of mHealth- pilot studies, there has been insufficient programmatic evidence to inform implementation and scale-up of mHealth as there are a few interventions in resource limited settings that assess scalability and sustainability of the digital interventions [17].

The evaluation of digital health systems and intervention is mainly focused on objective assessment of intervention with the aim of determining efficiency, effectiveness and impact [18].

For mHealth to achieve its potential, health apps need to be tailored to user accessibility and health needs, and more understanding of what hinders frequent users of digital technologies [19]**.**

Researcher- driven technologies often don’t go beyond pilot research studies, between 2008–2009, in Uganda 23 of 36 studies never went beyond pilot phase [17], the failure in assessing sustainability and scalability during the pilot trial in the long run creates the issue of pilotitis.

To ensure sustainability, m-health programmes must have strategic goals that are aligned with those of the national health and education system, and the initiatives must be owned and led by local stakeholders.

Previous studies have identified issues such as: acceptability to patients, costs, usability as barriers to implementation of mHealth [20], however the same study, identified public and professional willingness to mHealth as enablers of implementation and scalability.

In our study, over 95% (478) of study participants were willing to have continued mHealth adherence support, the participants who were on the mHealth Intervention were four times more willing to continue with the support, unlike the participants on standard of care arm, this is not surprising, since the participants on the intervention had prior experience and had benefitted from the intervention. The proportion willing to have mHealth was higher in our community compared to one in Ethiopia, where willingness was at 70.5%, however, the Ethiopian study was with Diabetes while our population was in HIV [21].

In North West Ethiopia, another study of HIV population, willingness to receive text message medication reminders, was only 50.9%, this is way below our number despite the population being similar, the reason for the high number in our study may be probably due to the use of interactive voice calls and secret pin codes which ensured privacy and making the participants safe to use the support [22].

In the rural United States, a 24-Survey respondents of whom 63% were in good health, a total of 65%, were willing to receive prerecorded messages for appointment reminders from the doctor [23].

In rural India, the willingness to receive mobile-phone based reminders is similar to our findings, and they noted that receiving reminders for drug adherence was acceptable to most 479 (98%) of respondents, 424 (89%) preferred voice calls alone to other forms of communication [24].

User pay-for-service means that the user pays for all or a portion of the cost of the service.

they receive “co-pay”. In the context of an SMS service, users can pay for messages that they send and receive, pay subscription fees to receive or access messages from a specific content provider, or pay a tiered or premium cost for more valuable content which would subsidize free content [25], for the purpose of C4LU, we assessed user- pay for C4LU system which provides pill reminder calls, health voice-messages, clinic appointment reminders as a whole package.

The goal of this study was to explore strategies for mHealth program sustainability and develop cost-recovery models for C4LU system. Evaluating willingness to pay is one mode of assessing sustainability of the services, and this shows the potential to scale-out the services to low-access settings, in our study, the participants on both arms expressed their willingness to pay for the services with over 65% of the participants expressing this willingness to pay, however the participants who were on intervention were almost twice as likely to pay as those on standard of care.

Whereas our study was assessing willingness to pay, another model in neighboring country Tanzania, used four scenarios to leverage strategic partnerships to reduce per-SMS program costs and create per-SMS program revenue and varied the structure for user financial contribution, in their results and they conducted break-even and analyses to evaluate the costs and revenues of these models, their results reveal that breaking even was only probable when all SMS costs were transferred to users and the lowest per-SMS cost was negotiated with telecom partners [25].

Majority of the study participants were willing to pay 1.35 USD monthly. The amount stated is comparable to the Indian program cost of USD $1.27-$1.77 per patient per year, and the projected total cost of the SMS reminder program from the Indian National AIDS Control Program (NACP) of sending mobile phone reminders to improve adherence to Antiretroviral Treatment (ART) among people living with HIV [26].

### Strengths and limitations

This was a mixed methods assessment and the qualitative findings backed up the quantitative findings from both urban and semi-urban population. The qualitative data was from the intervention arm participants which makes the data stronger as they gave feedback based on experiences with mHealth services. The ability to pay was probably based on individual’s income, which was not explored, thus making willingness to pay hypothetical***.***

## Conclusion

The benefits of the system outweighed the challenges evidenced by willingness to pay some reasonable amount for C4LU services. Payment for the service offers an opportunity to scale up and sustain mHealth in PLHIV in low resource settings, however, the amount mentioned by the participants does not support sustainability, however, the fees could be shared in a “Co-pay” model, and the amount can be apportioned based on the mhealth services offered.

## Supplementary Information

**Additional file 1**. Figure illustrates the system call flows.

**Additional file 2**. Willingness questionnaire administered at exit visit.

## Data Availability

All the necessary data has been submitted, however dataset is available on request on one by one case.

## References

[1] UNAIDS, FACT SHEET-Global AIDS UPDATE 2019. 2019.

[2] WHO, Consolidated guidelines on general HIV care and the use of antiretroviral drugs for treating and preventing HIV infection: recommendations for a public health approach. Geneva: World Health Organization, 2013: p. 269. 24716260

[3] Ministry of Health, U., Consolidated guidelines for prevention and treatment of HIV in Uganda. 2016.

[4] Akter S, Ray P (2010). mHealth-an ultimate platform to serve the unserved. Yearb Med Inf.

[5] Free C (2013). The effectiveness of mobile-health technology-based health behaviour change or disease management interventions for health care consumers: a systematic review. PLoS Med.

[6] Leon N, Schneider H, Daviaud E (2012). Applying a framework for assessing the health system challenges to scaling up mHealth in South Africa. BMC Med Inf Decis Mak.

[7] Campbell JI, Haberer JE (2015). Cell phone-based and adherence device technologies for HIV care and treatment in resource-limited settings: recent advances. Curr HIV/AIDS Rep.

[8] Lester RT (2010). Effects of a mobile phone short message service on antiretroviral treatment adherence in Kenya (WelTel Kenya1): a randomised trial. The Lancet.

[9] Haberer JE (2016). Short message service (SMS) reminders and real-time adherence monitoring improve antiretroviral therapy adherence in rural Uganda. AIDS (London, England).

[10] Ndlovu K (2014). Scaling up a mobile telemedicine solution in Botswana: keys to sustainability. Front Public Health.

[11] Finitsis DJ, Pellowski JA, Johnson BT (2014). Text message intervention designs to promote adherence to antiretroviral therapy (ART): a meta-analysis of randomized controlled trials. PLoS ONE.

[12] Muhambe TMO, Daniel O, Wagacha PW. Proposing parameters for evaluating sustainability of mHealth systems in developing countries. Int J Comput Technol. 17(1).

[13] Byonanebye DM (2021). An interactive voice response software to improve the quality of life of people living with HIV in Uganda: randomized controlled trial. JMIR mHealth uHealth.

[14] Agnes BN, Maria SN, Josephine A, Benson N, Elizabeth O, Noela CO, Agnes K, Barbara C, Andrew K, Rachel K, Elly K, Mohammed L, Rosalind P-R Sustainability of mHealth interventions: patients’ preferences and willingness to pay user fees for mHealth ART adherence support tool in resource limited settings: TUPED 683. In: IAS. 2019: Mexico City.

[15] Flämig K (2019). ART adherence clubs in the Western Cape of South Africa: What does the sustainability framework tell us? A scoping literature review. J Int AIDS Soc.

[16] Rasschaert F (2014). Sustainability of a community-based anti-retroviral care delivery model–a qualitative research study in Tete, Mozambique. J Int AIDS Soc.

[17] Tomlinson M (2013). Scaling Up mHealth: Where is the evidence?. PLOS Med.

[18] WHO. Monitoring-and-Evaluating-Digital-Health-Interventions : A practical guide to conducting research and assessment. 2016.

[19] Somers C (2019). Valuing mobile health: an open-ended contingent valuation survey of a national digital health program. JMIR Mhealth Uhealth.

[20] Lennon MR (2017). Readiness for delivering digital health at scale: lessons from a longitudinal qualitative evaluation of a national digital health innovation program in the United Kingdom. J Med Internet Res.

[21] Jemere AT (2019). Access to mobile phone and willingness to receive mHealth services among patients with diabetes in Northwest Ethiopia: a cross-sectional study. BMJ Open.

[22] Kebede M (2015). Willingness to receive text message medication reminders among patients on antiretroviral treatment in North West Ethiopia: a cross-sectional study. BMC Med Inf Decis Mak.

[23] Sankaranarayanan J, Sallach RE (2014). Rural patients' access to mobile phones and willingness to receive mobile phone-based pharmacy and other health technology services: a pilot study. Telemed J E Health.

[24] DeSouza SI (2014). Mobile phones: the next step towards healthcare delivery in rural India?. PLoS ONE.

[25] Mangone ER (2016). Sustainable cost models for mHealth at Scale: modeling program data from m4RH Tanzania. PLoS ONE.

[26] Rodrigues R (2014). Mobile phones to support adherence to antiretroviral therapy: what would it cost the Indian National AIDS Control Programme?. J Int AIDS Soc.

