# Supplementary figures and images for: Willingness to pay for an mHealth anti-retroviral therapy adherence and information tool: Transitioning to sustainability, Call for life randomised study experience in Uganda
Source: BMC Med Inform Decis Mak. 2022 Feb 26;22:52. doi: 10.1186/s12911-022-01782-0 (PMC8882291; doi:10.1186/s12911-022-01782-0)

**Figure illustration of the call for life system call flows**

***
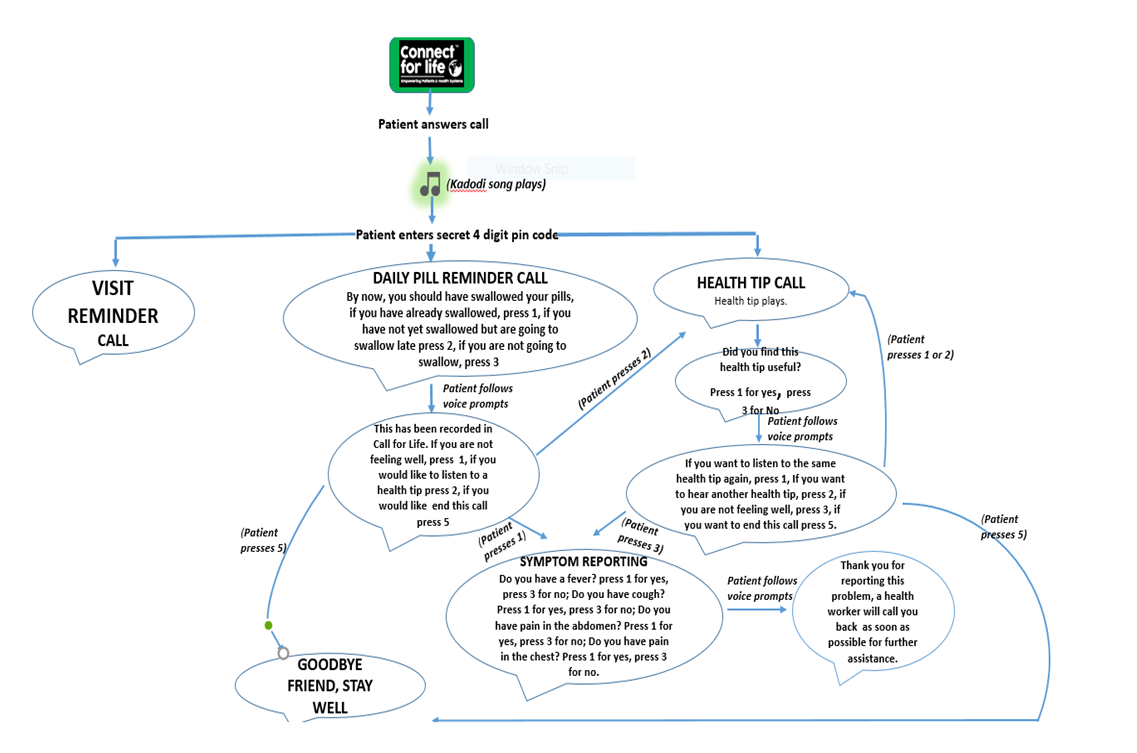
***

Supplement: Supplementary file 1 — Additional file 1. Figure illustrates the system call flows. [file 12911_2022_1782_MOESM1_ESM.docx]
