# Supplementary material for: Willingness to pay for an mHealth anti-retroviral therapy adherence and information tool: Transitioning to sustainability, Call for life randomised study experience in Uganda
Source: BMC Med Inform Decis Mak. 2022 Feb 26;22:52. doi: 10.1186/s12911-022-01782-0 (PMC8882291; doi:10.1186/s12911-022-01782-0)
Supplement: Supplementary file 2 — Additional file 2. Willingness questionnaire administered at exit visit. [file 12911_2022_1782_MOESM2_ESM.pdf]

CALL4LIFE(068) Willingness-1 (050)

STUDY ID

Visit # __ __ . __

__ - __ __ __ __ __

Site # Subject ID #

Clinic #: __ __ __ __ __

WILLINGNESS FORM

__ __ / __ __ / 2 0 __ __

dd mm yyyy

Instructions: Please administer this questionnaire at the Month 18 study visit and 24 Month or at last study visit (Exit Visit)

A.Type of adherence support

1. Having been on the C4L study, which arm of the study were you on?

- [ ] Active arm (receiving C4L tool and standard of care)
- [ ] Placebo (receiving Standard of Care)

B. Choice for the adherence support

2. Do you want the adherence support after the end of the study? (Chose one best one option)

- [ ] Yes
- [ ] No

2b. If Yes /No, why? ______________________________

3. How do you wish the support to be offered? (Choose the best option)

- [ ] Once a week
- [ ] Once a day
- [ ] Based on the pill reminder
- [ ] On Clinic Visits

Makerere University School of Public Health • Higher Degrees, Research and Ethics Committee • Valid Thru: 03 JUL 2018

3b. For any options above, state the reason why ______________________

C. Recommendation for the services

4. Would you recommend the adherence support services to a colleague?

- [ ] Yes
- [ ] No

4b. If Yes / No, Why? ______________________

5. Would you recommend the Government/ Ministry of Health to adapt the services for?

- [ ] Hypertensive Patients
- [ ] Diabetic Patients
- [ ] Patient on Tuberculosis treatment
- [ ] Other ______________________

CALL4LIFE(068) Willingness-2 (051)

STUDY ID

☐ - ☐☐☐☐☐
Site # Subject ID #

Clinic #: ☐☐☐☐☐

WILLINGNESS FORM

D.Rating of the services

6. which adherence support were you receiving? Choose all that apply

- ☐ IDI-PCT adherence support
- ☐ C4I-Study Team Adherence support
- ☐ C4L-Mobile Phone based adherence tool
- ☐ None **(If none stop here)**

7. With a scale of 1-5(5 Excellent, 1 Poor), how would you rate the adherence support you had past year

- ☐ 1:Poor
- ☐ 2:Fair
- ☐ 3:Good
- ☐ 4:Very good
- ☐ 5: Excellent

E. Willingness to pay for adherence support

8. If we offered everyone the tool but they had to pay for it, would you be willing to pay for the support.

☐ Yes ☐ No

9. How much would you like to pay for the services.

State the amount ____________________

10. How often would you pay for the services

- ☐ Daily ☐ Weekly
- ☐ Monthly ☐ Yearly
- ☐ Other (Specify) ____________________

Makerere University School of Public Health • Higher Degrees, Research and Ethics Committee • Valid Thru: 03 JUL 2018

11. How would you like the payment to be?

- ☐ Through mobile money as a top up depending on how much I use it
- ☐ As a fixed monthly service fee deduction from airtime
